# Systematic Review of Privacy-Preserving Distributed Machine Learning From Federated Databases in Health Care

**DOI:** 10.1200/CCI.19.00047

**Published:** 2020-03-05

**Authors:** Fadila Zerka, Samir Barakat, Sean Walsh, Marta Bogowicz, Ralph T. H. Leijenaar, Arthur Jochems, Benjamin Miraglio, David Townend, Philippe Lambin

**Affiliations:** ^1^ The D-Lab, Department of Precision Medicine, GROW School for Oncology and Developmental Biology, Maastricht University Medical Centre, Maastricht, The Netherlands; ^2^ Oncoradiomics, Liège, Belgium; ^3^ Department of Radiation Oncology, University Hospital Zurich and University of Zurich, Zurich, Switzerland; ^4^ Department of Health, Ethics, and Society, CAPHRI (Care and Public Health Research Institute), Maastricht University, Maastricht, The Netherlands

## Abstract

Big data for health care is one of the potential solutions to deal with the numerous challenges of health care, such as rising cost, aging population, precision medicine, universal health coverage, and the increase of noncommunicable diseases. However, data centralization for big data raises privacy and regulatory concerns.

Covered topics include (1) an introduction to privacy of patient data and distributed learning as a potential solution to preserving these data, a description of the legal context for patient data research, and a definition of machine/deep learning concepts; (2) a presentation of the adopted review protocol; (3) a presentation of the search results; and (4) a discussion of the findings, limitations of the review, and future perspectives.

Distributed learning from federated databases makes data centralization unnecessary. Distributed algorithms iteratively analyze separate databases, essentially sharing research questions and answers between databases instead of sharing the data. In other words, one can learn from separate and isolated datasets without patient data ever leaving the individual clinical institutes.

Distributed learning promises great potential to facilitate big data for medical application, in particular for international consortiums. Our purpose is to review the major implementations of distributed learning in health care.

## INTRODUCTION

Law and ethics seek to produce a governance framework for the processing of patient data that produces a solution to the issues that arise between the competing desires of individuals in society for privacy and advances in health care. Traditional safeguards to achieve this governance have come from, for example, the anonymization of data or informed consent. These are not adequate safeguards for the new big data and artificial intelligence methodologies in research; it is increasingly difficult to create anonymous data (rather than pseudonymized/coded data) or to maintain it against re-identification (through linking of datasets causing accidental or deliberate re-identification). The technology of big data and artificial intelligence, however, itself increasingly offers safeguards to solve the governance problem. In this article we explore how privacy-preserving distributed machine learning from federated databases might assist governance in health care. The article first outlines the basic parameters of the law and ethics issues and then discusses machine learning and deep learning. Thereafter, the results of the review are presented and discussed. The methodology for this research is that distributed machine learning is an evolving field in computing, with 665 articles published between 2001 and 2018; the study is based on a literature search, focuses on the medical applications of distributed machine learning, and provides an up-to-date summary of the field.

## THE LEGAL CONTEXT FOR PATIENT DATA RESEARCH

The challenges in law and ethics in relation to big data and artificial intelligence are well documented and discussed^1-16^. The issue is one of balance: privacy of health data and access to data for research. This issue is likely to become more pronounced with the foreseeable developments in health care, notably in relation to rising cost, aging population, precision medicine, universal health coverage, and the increase of noncommunicable diseases. However, recent developments in law, for example, in the European Union’s General Data Protection Regulation (GDPR), appear to maintain the traditional approach that seems to favor individualism above solidarity. Individualism is strengthened in the new legislation. There is a narrowing of the definition of informed consent in Article 4.11 of the GDPR, with the unclear inclusion of the necessity for broad consent in scientific research included in Recital 33.

**CONTEXT**

**Key Objective**
Review the contribution of distributed learning to preserve data privacy in health care.
**Knowledge Generated**
Data in health care are greatly protected; therefore, accessing medical data is restricted by law and ethics. This restriction has led to a change in research practice to adapt to new regulations. Distributed learning makes it possible to learn from medical data without these data ever leaving the medical institutions.
**Relevance**
Distributed learning allows learning from medical data while guaranteeing preservation of patient privacy.

In relation to the continuing ambiguity of the unclear legal landscape for research using and reusing large datasets and linking between datasets, the GDPR is not clear in the area of re-identification of individuals. For the GDPR, part of the problem is clear—when data have the potential when added to other data to identify an individual, then those data are personal data and subject to regulation. The question is, is this absolute (any possibility, regardless of remoteness), or is there a reasonableness test? Recital 26 includes such a reasonable test: “To ascertain whether means are reasonably likely to be used to identify the natural person, account should be taken of all objective factors, such as the costs of and the amount of time required for identification, taking into consideration the available technology at the time of the processing and technological developments.”^16a^

From this overview of legal difficulties, it is clear that there are obstacles to processing data in big data, machine learning, and artificial intelligence methodologies and environments. It must be stressed that the object is not to circumvent the rights of patients or to suggest that privacy should be ignored. The difficulty is that where the law is unclear, there is a tendency toward restrictive readings of the law to avoid liability, and, in the case of the methodologies and applications of data science discussed here, the effect of unclear law and restrictive interpretations of the law will be to block potentially important medical and scientific developments and research. Each of the uncertainties will require regulators to take a position on the best interpretation of the meaning of the law according to the available safeguards. The question for the data science community is, how far can that community itself address concerns about privacy, about re-identification, and about safeguarding autonomy of individuals and their legitimate expectations to dignity in their treatment through the proper treatment of their personal data? How far distributed learning might contribute a suitable safeguard is the question addressed in the remainder of this paper.

## MACHINE LEARNING

Machine learning comes from the possibility to apply algorithms on raw data to acquire knowledge.^1^ These algorithms are implemented to support decision making in different domains, including health care, manufacturing, education, financial modeling, and marketing.^2,3^ In medical disciplines, machine learning has contributed to improving the efficiency of clinical trials and decision-making processes. Some examples of machine learning applications in medicine are the localization of thoracic diseases,^4^ early diagnosis of Alzheimer disease,^5^ personalized treatment,^6^ outcome prediction,^7,8^ and automated radiology reports.^9^

There are three main categories of machine learning algorithms. First, in supervised learning, the algorithm generates a function for mapping input variables to output variables. In unsupervised learning, the applied algorithms do not have any outcome variable to estimate, and the algorithms generate a function mapping for the structure of the data. The third type is referred to as reinforcement learning, whereby in the absence of a training dataset the algorithm trains itself by learning from experiences to make increasingly improved decisions. A reinforcement agent decides what action to perform to accomplish a given task.^10,11^ Table 1 provides a brief description of selected popular machine learning algorithms across the three categories.

**TABLE 1.**
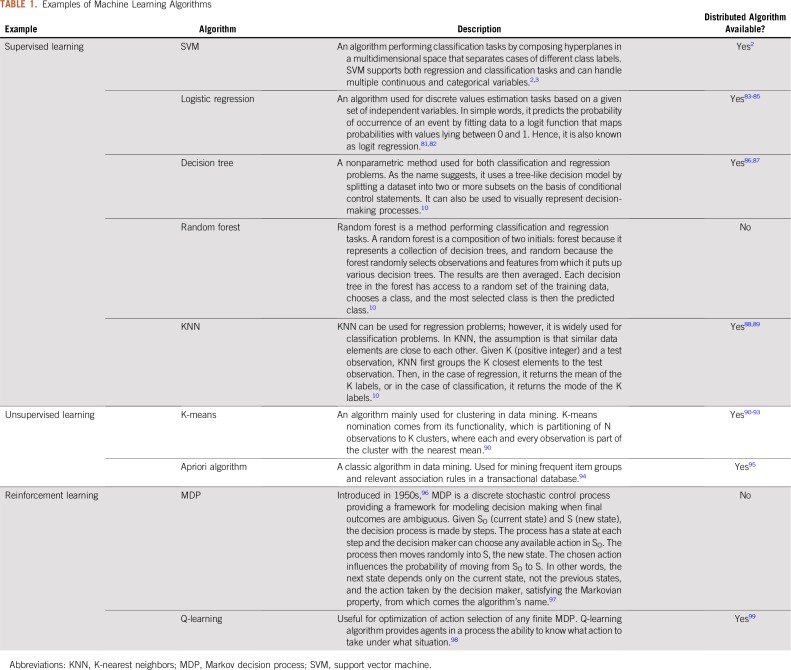
Examples of Machine Learning Algorithms

## DEEP LEARNING

**FIG 1.**
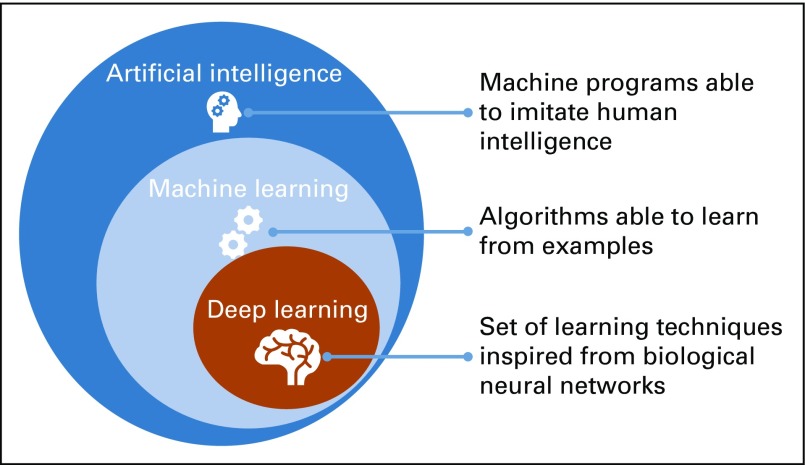
Relationship between artificial intelligence, machine learning, and deep learning.

Deep learning is a subset of machine learning, which, in turn, is a subset of artificial intelligence,^12^ as represented in Figure 1. The learning process of a deep neural network architecture cascades through multiple nodes in multiple layers, where nodes and layers use the output of the previous nodes and layers as input.^13^ The output of a node is calculated by applying an activation function to the weighted average of this node’s input. As described by Andrew Ng^14^, “The analogy to deep learning is that the rocket engine is the deep learning models and the fuel is the huge amounts of data that we can feed in to these algorithms,” meaning that the more data are fed into the model the better the performance. Yet, this continuous improvement of the performance in concordance with the amount of the data are not correct for traditional machine learning algorithms reaching a steady performance level that does not improve with the increase of the amount of the training data.^15^

## METHODS AND MATERIAL SELECTION

**FIG A1.**
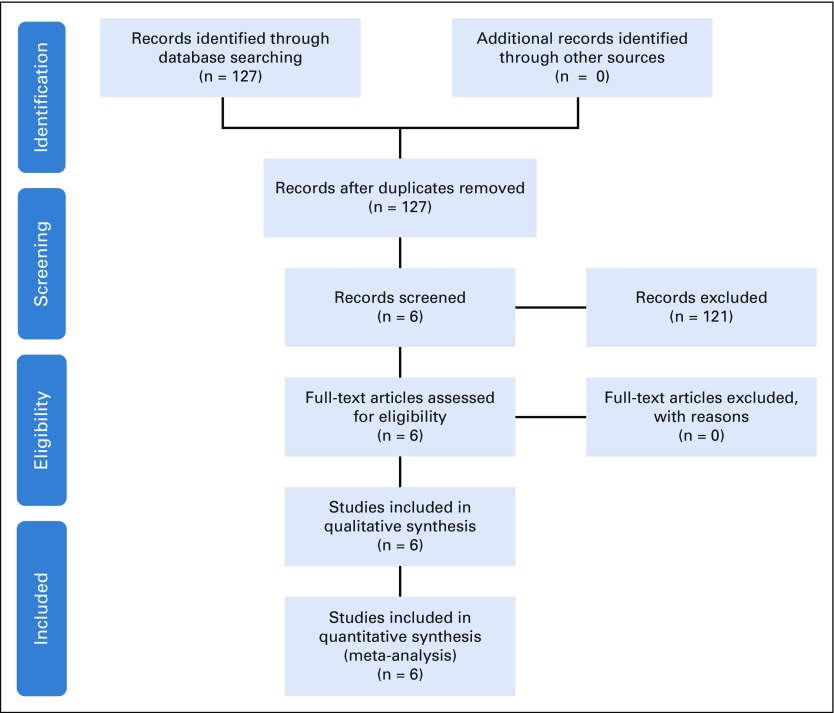
Preferred Reporting Items for Systematic Reviews and Meta-Analyses (PRISMA) 2009 flow diagram.

A PubMed search was performed to collect relevant studies concerning the utilization of distributed machine learning in medicine. We used the search strings: “distributed learning,” “distributed machine learning,” and “privacy preserving data mining.” The Preferred Reporting Items for Systematic Reviews and Meta-Analyses (PRISMA) statement was adopted to select and compare distributed learning literature.^16^ The PRISMA flow diagram and checklist are slightly modified and presented in Appendix Figure A1 and Appendix Table A1, respectively. The last search for distributed machine learning articles was performed on February 28, 2019.

## SEARCH RESULTS

A total of 127 articles were identified in PubMed using the search query: (“distributed learning” OR “distributed machine learning” OR “privacy preserving data mining”). Six papers were screened; a brief summary of each article is presented in Table 2.

**TABLE 2.**
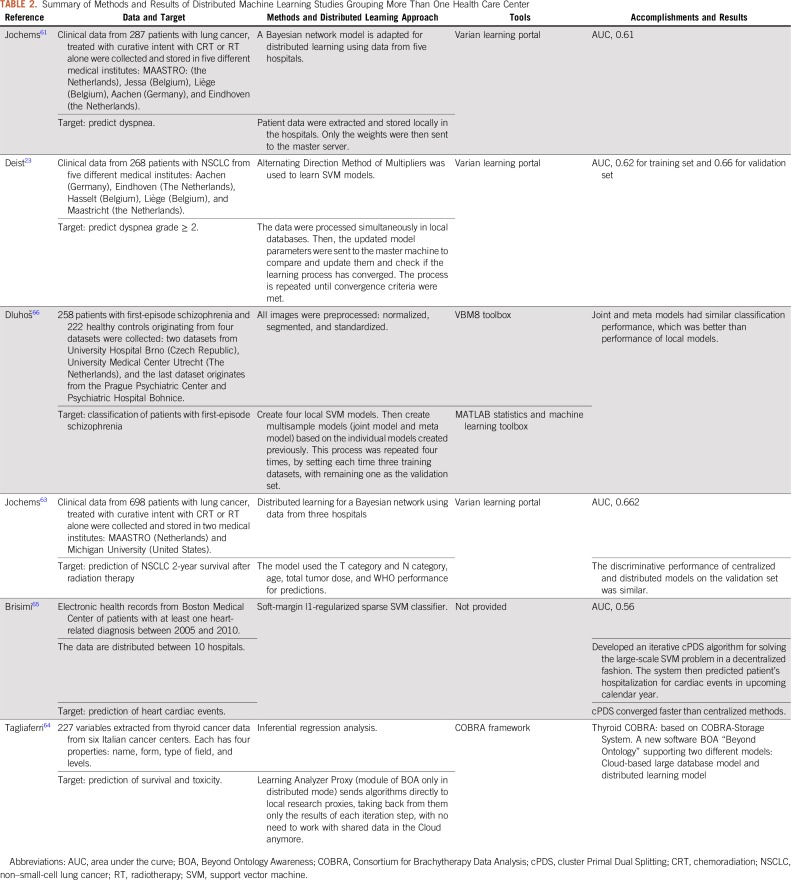
Summary of Methods and Results of Distributed Machine Learning Studies Grouping More Than One Health Care Center

## DISTRIBUTED LEARNING

**FIG 2.**
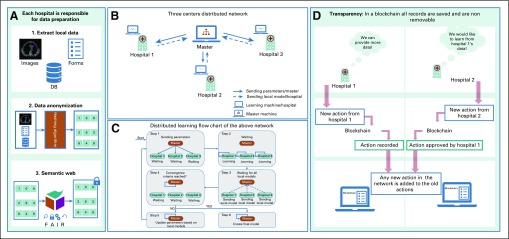
Schematic representation of the processes in a transparent distributed learning network. (A) Data preparation steps. (B) Distributed learning network, which is composed of three hospitals, each of which is equipped with a learning machine that can communicate with a master machine responsible for sending model parameters and checking convergence criteria. (C) Flowchart of the distributed learning network described in B. (D) Example of an action that can be tracked by blockchain (designed and implemented according to needs agreed among network members) and keep all network participants aware of any new activity taken in the network. DB, database; FAIR, findable, accessible, interoperable, reusable.

Distributed learning ensures data safety by only sharing mathematical parameters (or metadata) and not the actual data or in any instance data that might enable tracking back the patient information (such as patient ID, name, or date of birth). In other words, distributed algorithms iteratively analyze separate databases and return the same solution as if data were centralized, essentially sharing research questions and answers between databases instead of data.^17^ Also, before processing with the learning process, researchers must make sure all data have been successfully anonymized and secured by means of hashing algorithms and semantic web techniques, respectively, as can be seen in Figure 2, in addition to post-processing methods to address the multicenter variabilities.^19^

### Distributed Machine Learning

A large quantity of training data is required for machine learning to be applied, especially in outcome modeling, where multiple factors influence learning. Provided there are sufficient and appropriate data, machine learning typically results in accurate and generalizable models.^20,21^ However, the sensitivity of the personal data greatly hinders the conventional centralized approach to machine learning, whereby all data are gathered in a single data store. Distributed machine learning resolves legal and ethical privacy concerns by learning without the personal data ever leaving the firewall of the medical centers.^22^

The euroCAT^23^ and ukCAT^24^ projects are a proof of distributed learning being successfully implemented into clinical settings to overcome data access restrictions. The purpose of the euroCAT project was to predict patient outcomes (eg, post-radiotherapy dyspnea for patients with lung cancer) by learning from data stored within clinics without sharing any of the medical data.

### Distributed Deep Learning

Training a deep learning model typically requires thousands to millions of data points and is therefore computationally expensive as well as time consuming. These challenges can be mitigated with different approaches. First, because it is possible to train deep learning models in a parallelized fashion,^25^ using dedicated hardware (graphics processing units, tensor processing units)^26^ reduces the computational time. Second, as the memory of this dedicated hardware is often limited, it is possible to divide the training data into subsets called batches. In this situation, the training process iterates over the batches, only considering the data of one batch at each iteration.^27^ On top of easing the computing burden, using small batches during training improves the model’s ability to generalize.^28^

These approaches address computation challenges but do not necessarily preserve data privacy. As for machine learning, deep learning can be distributed to protect patient data.^29,30^ Moreover, distributed deep learning also improves computing performance, as in the case of wireless sensor networks, where centralized learning is inefficient in terms of both communication and energy.^31,32^

An example of distributed deep learning in the medical domain is that of Chang et al,^33^ who deployed a deep learning model across four medical institutions for image classification purposes using three distinct datasets: retinal fundus, mammography, and ImageNet. The results were compared with the same deep learning model trained on centrally hosted data. The comparison showed that the distributed model accuracy is similar to the centrally hosted model.^33^ In a different study, McClure et al^34^ developed a distributed deep neural network model to reproduce FreeSurfer brain segmentation. FreeSurfer is an open source tool for preprocessing and analyzing (segmentation, thickness estimation, and so on) of human brain magnetic resonance images.^35^ The results demonstrated performance improvement on the test datasets. Similar to the previous study, a brain tumor segmentation was successfully performed using distributed deep learning across 10 institutions (BraTS distribution).^36^

In the matter of distributed deep learning, the training weights are combined to train a final model, and the raw data are never exposed.^35,37^ In the case of sharing the locale gradients,^25^ it might be possible to retrieve estimations of the original data from these gradients. Training the local models on batches may prevent retrieving all the data from the gradients, as these gradients correspond to single batches rather than all the local data.^38^ However, setting an optimal batch size needs to be considered^25^ to assure data safety and the model’s ability to generalize.^28,39,40^

## PRIVACY AND INTEGRATION OF DISTRIBUTED LEARNING NETWORKS

Privacy in a distributed learning network addresses three main areas: data privacy, the implemented model’s privacy, and the model’s output privacy. Data privacy is achieved by means of data anonymization and data never leaving the medical institutions. The distributed learning model can be secured by applying differential privacy techniques,^41^ preventing leakage of weights during the training, and cryptographic techniques.^42^ These cryptographic techniques provide a set of multiparty protocols that ensure security of the computations and communication. Once the model is ready, not only can the network participants use it to learn from their data, but this learning should be able to be performed locally and under highly private and secure conditions to protect the model’s output.^23^

The users of a machine/deep learning model are not necessarily the model’s developers. Hence, documentation and the integration of automated data eligibility tests have two important assets:

- The documentation ensures providing a clear view of what the model is designed for, a technical description of the model, and its use.
- The eligibility tests are important to ensure that correct input data are extracted and provided before executing the model. In euroCAT,^23^ a distributed learning expert installed quality control via data extraction pipelines at every participant point in the network. The pipeline automatically allowed data records fulfilling the model training eligibility criteria to be used in the training. The experts also test the extraction pipeline thoroughly in addition to the machine learning testing. However, there were post-processing compensation methods to correct for the variations caused by using different local protocols.^19^

## DISCUSSION

If one examines oncology, for instance, cancer is clearly one of the greatest challenges facing health care. More than 16 million new cancer cases were reported in 2017 alone.^43^ This number climbed to 18.1 million cases in 2018.^44^ This increasing number of cancer incidences^45^ means that there are undoubtedly sufficient data worldwide to put machine/deep learning to meaningful work. However, as highlighted earlier, this requires access to the data and, as also highlighted earlier, distributed learning enables this in a manner that resolves legal and ethical concerns. Nonetheless, integration of distributed learning into health care is much slower compared with other fields, which raises the question of why this should be. Here, we summarize a set of methodologies to facilitate the adoption of distributed learning and provide future directions.

## CURRENT STATE OF MEDICAL DATA STORAGE AND PREPROCESSING

### Information Communication Technology

Every hospital has its own storage devices and architecture.^38,39^ In this case, the information communication technology preparation for distributed learning requires significant energy, time, and manpower, which can be costly. This same process (data acquisition and preprocessing) needs to be repeated for each participating hospital,^46-48^ and subsequently development and adoption of medical data standardization protocols need to be developed for this implementation process.

### Make the Data Readable: Findable, Accessible, Interoperable, Reusable Data Principles

**FIG 3.**
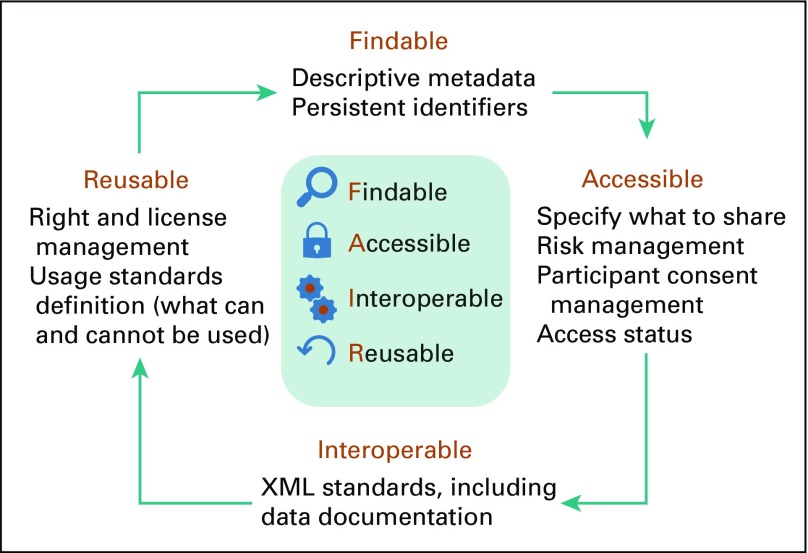
Description of findable, accessible, interoperable, reusable (FAIR) principles.

One way to enable a virtuous circle network effect is to embrace another community engaged in synergistic activities (joining a distributed learning network is worthwhile if it links to another large network). The Findable, Accessible, Interoperable, Reusable (FAIR) Guiding Principles for data management and stewardship have gained substantial interest, but delivering scientific protocols and workflows that are aligned with these principles is significant.^49^ A description of FAIR principles is represented in Figure 3. Technological solutions are urgently needed that will enable researchers to explore, consume, and produce FAIR data in a reliable and efficient manner, to publish and reuse computational workflows, and to define and share scientific protocols as workflow templates.^50^ Such solutions will address emerging concerns about the nonreproducibility of scientific research, particularly in data science (eg, poorly published data, incomplete workflow descriptions, limited ability to perform meta-analyses, and an overall lack of reproducibility).^51,52^ Because workflows are fundamental to research activities, FAIR has broad applicability, which is vital in the context of distributed learning with medical data.

## WHY NOT PUBLICLY SHARE MEDICAL DATA?

Some studies were conducted trying to facilitate and secure data-sharing procedures to encourage related researchers and organizations to publicly share their data and embrace transparency,^53^ by proposing data-sharing procedures and protocols aiming to harmonize regulatory frameworks and research governance.^54,55^ Despite the efforts made toward data-sharing globalization, the sociocultural issues surrounding data sharing remain pertinent.^56^ Large clinical trials also face limitations in the data collection capabilities because of limited data storage capacities and manpower. To retrospectively perform additional analysis, all the participating centers need to be contacted again, which is time consuming and delays research.^57^

Furthermore, medical institutions prefer not to share patient data to ensure privacy protection.^58^ This is, of course, in no small part about ensuring the trust and confidence of patients who display a wide range of sensitivities toward the use of their personal data.

## ORGANIZATIONAL CHANGE MANAGEMENT

The adoption of distributed learning will require a change in organizational management (such as making use of newest data standardization techniques and adapting the roles of employees to more technically oriented tasks, such as data retrieval). Provided knowledge and understanding of proper change management concepts, health care providers can implement the latter successfully.^59^ Change management principles, such as defining a global vision, networking, and continuous communicating, could facilitate the integration of new technologies and bring up the clinical capabilities. However, this process of change management can be complicated, because it requires the involvement of multiple health care centers from different countries and continents. This diversity can trigger a fear of loss (one of the major factors of financial decision making), which stems from differences of opinion and regulation,^60^ and the absence of data standardization, making the processes of data acquisition and preprocessing harder. In addition, the lack of knowledge about the new technology leads to resistance to accept the change and innovation.^60,61^ Therefore, it is important to help health care organizations understand the need for distributed learning by explaining the context of the change in terms of traditional ways of learning to distributed learning and a long-term vision of the improvements that it can bring, including time and money savings for both hospitals and patients. This could in turn improve patient lives, in addition to conducting more studies on research databases to consolidate proof of safety and quality of distributed models.

As can be seen in Table 2, distributed learning has been applied to train different models that can predict different outcomes for a variety of pathologies, including lung cancer,^23,62,63,63a^ thyroid cancer,^64^ heart cardiac events,^65^ and schizophrenia,^66^ in addition to the continuous development of tools and algorithms facilitating the adoption of distributed learning, such as the variant learning portal, the alternating direction method of multipliers algorithm,^2^ as well as the application of FAIR data principles. The cited studies provide a proof that distributed learning can ensure patient data privacy and guarantee that accurate models are built that are the equivalent of centralized models.

## LIMITATIONS OF THE EXISTING DISTRIBUTED LEARNING IMPLEMENTATIONS

A shared limitation of the studies presented in Table 2 is that the number of institutes involved in the distributed network is rather small. The size of the network varies from four to 10 institutions. With few medical institutes involved, the models were trained using the data of only a few hundred patients. By promoting the use of distributed learning, it should instead be possible to train the models using data from thousands or even millions of patients.

## FUTURE PERSPECTIVES

An automated monitoring system accessible by the partners or medical centers participating in the distributed learning network can promote transparency, traceability, and trust.^67^ Recent advances of information technology, such as blockchain, can be integrated into a distributed learning network.^68^ Blockchain allows trusted partners to visualize the history of the transactions and actions taken in the distributed network. This integration of blockchain should help in easing the resistance to the new distributed technology among health care workers as it provides both provenance and enforceable governance.

**FIG 4.**
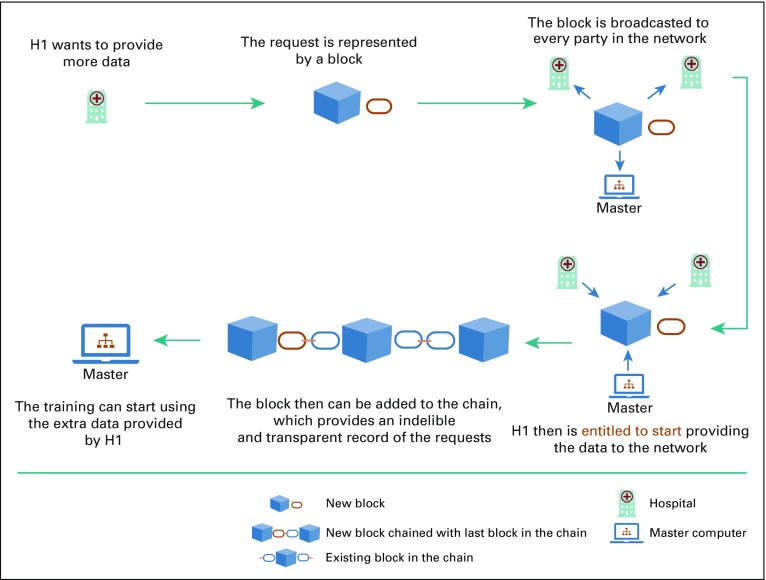
Visual representation of blockchain. Adapted from Rennock et al.^18^

In 2008, Satoshi Nakamoto^69^ introduced the concept of a peer-to-peer electronic cash system known as Bitcoin. Blockchain was made famous as the public transaction ledger of this cryptocurrency.^69,70^ It ensures security by using cryptography in a decentralized, immutable distributed ledger technology.^71^ It is easy to manage as it can be made public, whereby any individual can participate, or it can be made private, where all participants are known to each other.^72^ It is an efficient monitoring system, as records cannot be deleted from the chain. By these means, blockchain exceeds its application as a cryptocurrency to a permanent trustful tracing system. Figure 4 illustrates a visual representation of blockchain.

Boulos et al^71^ demonstrated how blockchain could be used to contribute in health care: securing patient information and provider identities, managing health supply chains, monetizing clinical research and data (giving patients the choice to share), processing claims, detecting fraud, and managing prescriptions (replace incorrect and outdated data). In addition to the above-mentioned uses of blockchain, it has been also used to maintain security and scalability of clinical data sharing,^73^ secure medical record sharing,^74^ prevent drug counterfeiting,^75^ and secure a patient’s location.^76^

It is essential that the use of distributed machine/deep learning and blockchain be harmonized with the available security-preserving technologies (ie, continues development and cybersecurity), which begins at the user levels (use strong passwords, connect using only trusted networks, and so on) and ends with more complex information technology infrastructures (such as data anonymization and user ID encryption).^77^ Cybersecurity is a key aspect in preserving privacy and ensuring safety and trust among patients and health care systems.^78^ The continuous development or postmarketing surveillance can be seen as the set of checks and integrations that should occur when a distributed learning network is launched. This practice should make it possible to identify any weak security measures in the network or non-up-to-date features that may require re-implementation.^79,80^

The distributed learning and blockchain technologies presented here show that there are emerging data science solutions that begin to meet the concerns and shortcomings of the law. The problems of re-identification are greatly reduced and managed through the technologies. Clearly, there are conceptual issues of understanding the impact of these technologies on privacy, and the relationship between privacy and confidentiality, but there are significant technical developments for the regulators to consider that could answer a number of their concerns.

## SUMMARY

Currently, a combination of regulations and ethics makes it difficult to share data even for scientific research purposes. The issues relate to the legal basis for processing and anonymization. Specifically, there has been reluctance to move away from informed consent as the legal basis for processing toward processing in the public interest, and there are concerns about the re-identification of individuals where data are de-identified and then shared in aggregated environments. A solution could be to allow researchers to train their machine learning programs without the data ever having to leave the clinics, which in this paper we have established as distributed learning. This safe practice makes it possible to learn from medical data and can be applied across various medical disciplines. A limitation to its application, however, is that medical centers need to be convinced to participate in such practice, and regulators also need to know suitable safeguards have been established. Moreover, as can be seen in Table 2, even with the use of distributed learning, the size of the data pool learned from remains rather small. In the future, the integration of blockchain technology to distributed learning networks could be considered, as it ensures transparency and traceability while following FAIR data principles and can facilitate the implementation of distributed learning.

## Figures and Tables

**TABLE A2.**
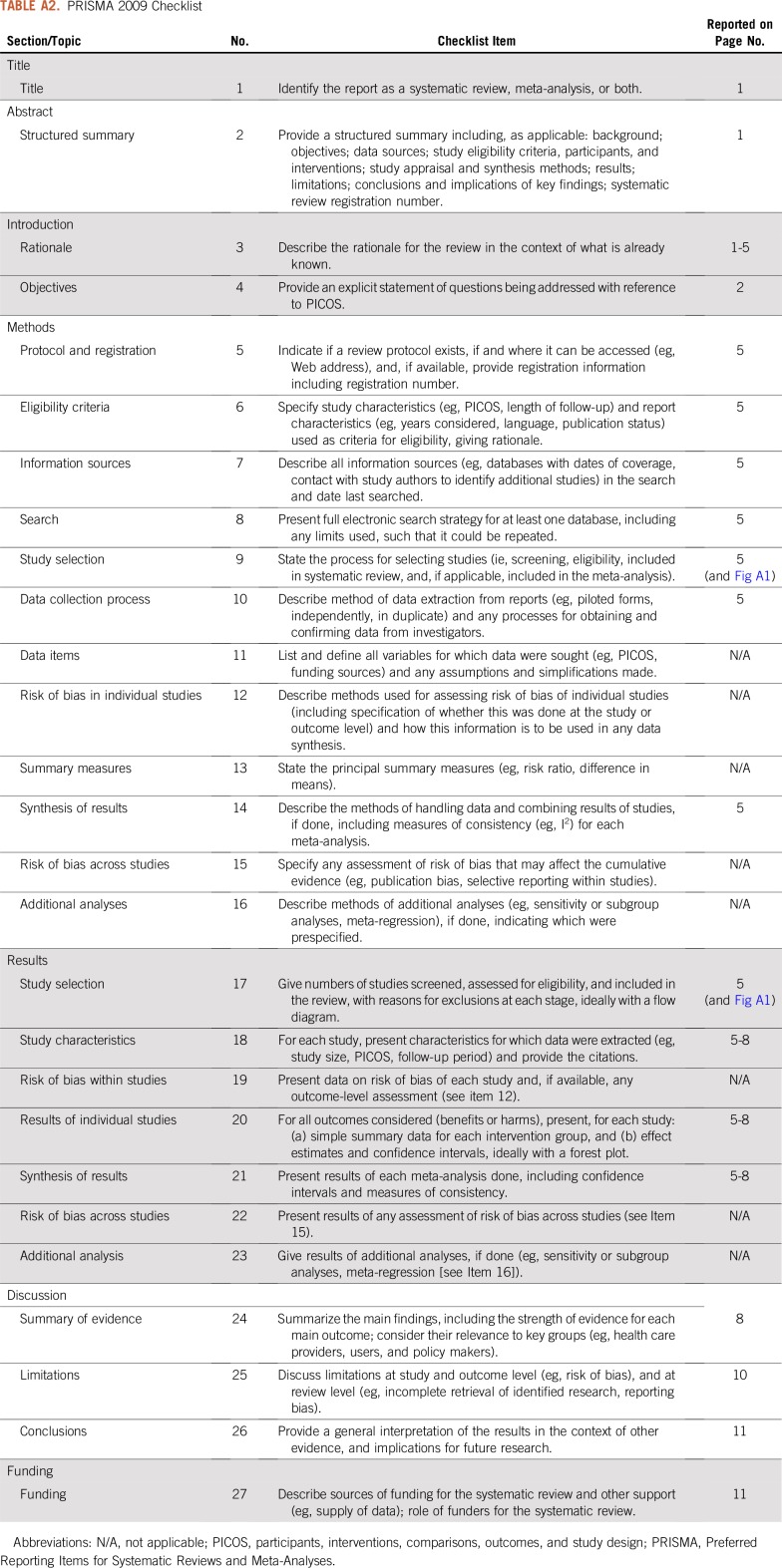
PRISMA 2009 Checklist
